# Conscientious use of patient-reported outcome measures in supportive care

**DOI:** 10.1007/s00520-023-07681-y

**Published:** 2023-03-22

**Authors:** Derek K. Smith

**Affiliations:** American Dental Association Science and Research Institute, Chicago, IL USA

**Keywords:** Patient-reported outcomes, Survey data, Statistical analysis, Power calculation

## Abstract

**Purpose:**

Patient-reported outcome measures (PRO) are critical tools to developing an understanding of cancer patients’ experience. This paper presents some of the lesser-understood implications of using patient-reported outcome measures in clinical research.

**Methods:**

This study uses a combination of literature sources, real-world examples from supportive care studies, and statistical simulations to demonstrate the operating characteristics of patient-reported measures.

**Results:**

It is demonstrated that care must be taken in the analysis of PROs as the assumptions of the most common mean-based approaches are often violated including linearity, normally distributed errors, interference with asymptotic convergence via boundary values, and more. Further, the implications of subjective discretization are shown to reduce the apparent statistical power of PRO-based studies.

**Conclusions:**

PRO-based studies must be designed conscientiously as each PRO item will demonstrate a varying degree of subjectivity in a given population. Sample sizes of randomized studies using PROs must be inflated to account for this. Analyses should consider using ordinal statistical models until such time as the assumptions of mean-based models can be verified.

## Introduction

With modern technological advances, it is easier than ever to administer patient-reported outcome measures (PRO) to increase surveillance of a patient’s condition [1]. This convenience combined with their versatility has led to PROs becoming a cornerstone of both clinical evaluation and research in the oncologic patient population [2, 3]. However, studies have suggested that the PRO measures are generally limited in their impact as a result of a lack of understanding in the community as to how these measures should be used to inform decision-making [4, 5] and a lack of disease-specific tools in oncology [6].

There is substantial evidence that PROs can positively impact clinical care [7–9]. One randomized study demonstrated the feasibility of PROs as a clinical tool that could lower psychological distress [9]. A second randomized study showed that health-related quality of life declined by less and that those oncologic patients participating in PRO-based monitoring visited the emergency department less frequently and were hospitalized less often [7]. Despite these demonstrations of potential efficacy, there is general agreement that further study in the implementation and interpretation spaces is warranted.

In addition to their utility as clinical adjuncts, PROs have become key research tools [2]. They have seen extensive utilization in the areas of adverse event reporting, palliative care, supportive care, and survivorship. The benefit of using PROs in the supportive setting is clear providing the ability to conduct more frequent surveillance at a lower cost. However, proper analysis and obtaining valid statistical inferences from PRO-based outcomes is substantially more challenging than it is with many clinically objective measures.

The increasing importance of PROs both clinically and as a research tool demands that members of the community obtain a better understanding of PROs and their treatment. In this manuscript, we detail common features and challenges associated with the interpretation and analysis of PROs.

## Introduction to patient-reported outcomes

Patient-reported outcome measures are defined broadly as any measure that is derived from the patient’s perception as opposed to objective clinical measurement or clinician report. Although the way this information is gathered can vary, most often PROs take the form of a questionnaire which has undergone an extensive methodological development. The rigor with which a tool is developed is often directly responsible for its utility, or lack thereof.

Although methods for tool development can vary by field and by intended use, it generally begins with a comprehensive literature review. Often insight can be gained from existing PROs in the literature that were developed to measure similar constructs. Constructs are aspects of the disease process or symptomatology that the PRO is intended to measure. Once various constructs are identified, a preliminary conceptual model is developed. The conceptual model often takes the form of a diagram that illustrates the interplay between various constructs.

The next step in PRO development is generally qualitative, taking the form of interviews or focus groups with patients living with the condition of interest. A well-performed qualitative study can identify additional constructs that are specific to a given disease process. In addition, the particular verbiage a patient uses to describe a symptom can be used in the questions meant to measure it. This can be important, especially in areas where health literacy is low or regional vernacular is common. Having abstracted a more comprehensive list of constructs from the qualitative study, the conceptual model can then be revisited and finalized.

With a complete conceptual model, it is then possible to develop a tool. The tool is then generally piloted in patients with the condition to assess redundancy of items (the degree to which responses to questions may be correlated), reliability (the degree to which patients give the same response under similar conditions), and various aspects of validity (the degree to which the tool measures the things it purports to measure). Rigorous development of a PRO helps ensure that the tool will suit its desired purpose and be applicable to the population in which it was developed.

The completed tool generally consists of several questions, which commonly are combined into subscales that represent the constructs identified in the conceptual model. There are many ways to combine individual items into a subscale; however, the most common way is to add or average the individual component items.

## Latent constructs

Generally, the goal of a PRO is to measure a latent construct. A latent construct is an aspect of the disease process which exists, but is expensive, inconvenient, or impossible to measure. Pain is an example of a latent construct. Theoretically, there are a set of biological processes that are quantifiable that are causing the patient to experience pain. However, quantifying pain requires these processes to be well-understood and readily measurable. Because the pathways are poorly understood, pain is often assessed via a visual analog scale.

Most PROs register the patient’s perception of their symptom severity on a discrete scale, often with five or ten levels. The 5-point Likert scale is a common example in which patients are asked to rate their level of agreement with a particular statement. This type of measurement can create challenges in analyzing and interpreting PRO data. Operating under the assumption that the PRO is intended as a surrogate for a continuous latent construct, the following sections detail some issues with analyzing PRO data.

### Subjective discretization

Discretization refers to the categorization of something that is naturally continuous. Discretization of PROs is generally subjective (meaning that the latent construct is categorized differently according to each patient’s perception of severity). For example, consider a PRO item that is asking about chemotherapy-associated hyposalivation. In this case, the latent construct that we are assessing could be related to salivary flow rate. A common PRO developed in head and neck cancer asks patients to respond to the prompt: “I have problems with dry mouth,” rating the severity on a scale from zero to ten. Each patient that the item is administered to may perceive the severity of salivary hypofunction differently. To illustrate, suppose two patients who both have a stimulated salivary flow rate of 0.75 ml/min are administered the item. Due to the severity being a function of the patients’ perception, one may rate the severity as a three and the other may rate it as a five. This is an example of subjective discretization, which is how the majority of PROs operate.

Discretization of any kind induces additional error in measuring the latent construct. Both the number of categories and the subjectivity of the cutoffs between categories affect the amount of measurement error induced. The effect of discretization can be limited by implementing PROs with large numbers of categories, i.e. a slider with 0–100 responses as opposed to a 5-point Likert scale, though the scaling will still be subjective.

**Fig. 1:**
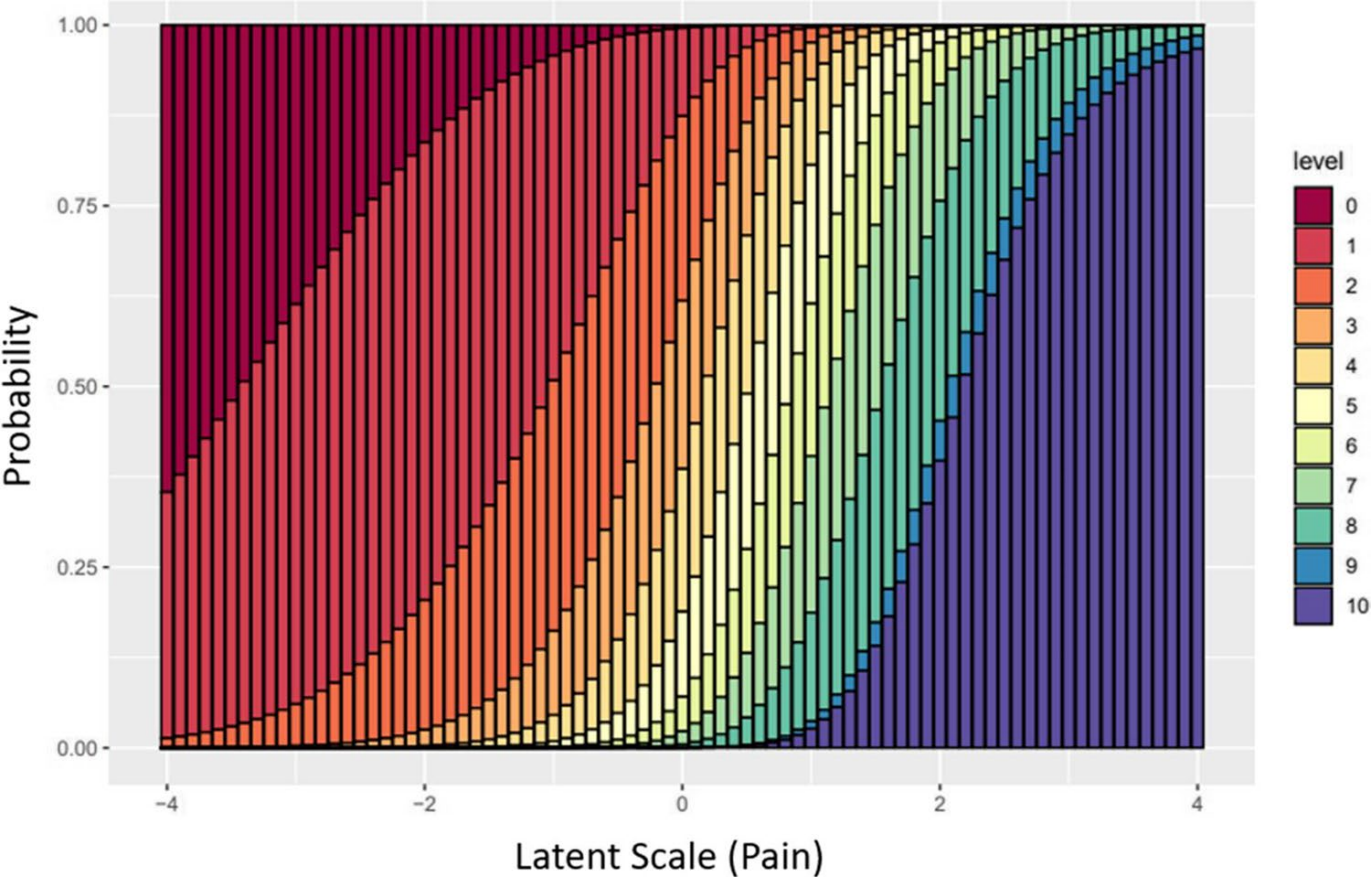
Results of Rasch analysis of the VHNSS pain subscale item 25 demonstrating that a given level of pain can elicit a wide variety of patient responses. This subjectivity introduces misclassification error resulting in conservative bias and a consummate decrease in statistical power

One way to visualize the subjectivity of a given PRO item with respect to the latent construct is by analyzing it with a Rasch model [10]. The Vanderbilt Head and Neck Symptom Survey’s (VHNSS) pain subscale, consists of 4 questions related to pain. Using the Rasch model, patient responses to all four of those questions can be used to estimate the patient’s most likely position on the latent scale and how each item in the subscale performs relative to that position (Fig. 1). In Fig. 1, we see a stacked bar chart with the patient’s true pain experience on the *x*-axis. The bars represent the probability that a patient with the level of pain indicated on the *x*-axis will respond to question 25 on the Vanderbilt Head and Neck Symptom Survey (VHNSS), “My average pain over the last weak has been…,” with each category from zero (no pain) to ten (severe pain). The figure demonstrates how our data suggests that patients with similar levels of pain have non-trivial probabilities of endorsing a wide variety of responses, particularly toward the middle of the pain scale. For example, at 0.5 on the *x*-axis, patients have a very similar probability of endorsing pain levels from 3 to 8.

### Statistical power and sample size

**Fig. 2:**
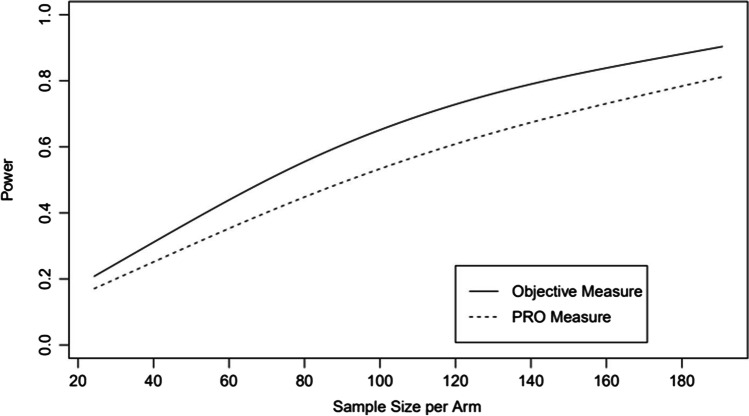
Results of statistical power simulation from a theoretical study using VHNSS item 25 as the primary outcome. The simulation shows that the subjectivity of the PRO responses results in a loss of statistical power which must be accounted for in the study design and complicates the interpretation of negative studies which fail to do so

This subjectivity in patient responses is a type of misclassification error. As with all misclassification errors, the result is that studies that use a PRO as the primary outcome measure will be inherently biased toward the null in comparison to a study with an objective measure. The degree of this bias will be dictated by the extent of the misclassification and be specific to each PRO item. To demonstrate this, consider a study in which we want to use the VHNSS item 25 as the primary outcome measure. In this study, there will be a control group and a treatment group whose pain values will be drawn from a distribution shifted 1/3^rd^ of a standard deviation (an approximate change of 0.9 units on the 0–10 scale), representing a mild treatment effect. If we ignore the bias introduced by the subjectivity in this particular PRO item, we would calculate that a sample size of roughly 145 patients per group would be sufficient. However, when we factor in the subjective patient responses, we find that we would actually need 185 patients per group to reach 80% power. The full simulation results are given in Fig. 2. For this reason, PRO-based studies that fail to account for PRO subjectivity in their design and analysis and subsequently fail to find an effect should be viewed with a degree of skepticism as they may have lacked adequate statistical power, even if calculations based on objective measures suggested that the sample size was adequate.

#### Boundary values

Boundary value problems arise from the constrained nature of PRO responses. For example, the Vanderbilt Head and Neck Symptom Survey asks patients to rate their symptom severity on a scale from zero to ten. Zero and ten would be referred to as boundaries. While latent constructs may or may not be bounded in fact, when patient responses are commonly near the boundaries, certain statistical methods can be adversely effected.

In a recent review of the statistical methods used to assess differences in PRO responses in breast cancer studies, the authors found that 24.6% of the studies had such an insufficient description of the analysis used that the reviewers could not determine which model was used. Among the articles in which the type of analysis was discernable, models that relied on assumptions of normality of the sampling distribution (*t*-test, ANOVA, and linear models) accounted for as much as 86.9% of the chosen statistical methods [11]. Unfortunately, methods that rely on assumptions of normality are generally poor approximations when utilized near a boundary. Recall from probability that the normal distribution on which these analyses are based is symmetric with tails that have significant probability density within 2 standard errors of the mean. If the mean response to a PRO item is within two standard errors of the boundary, it is impossible for the normal distribution to be a reasonable approximation. This can affect confidence interval coverage, as well as type I and type II error rates leading to spurious results.

**Fig. 3:**
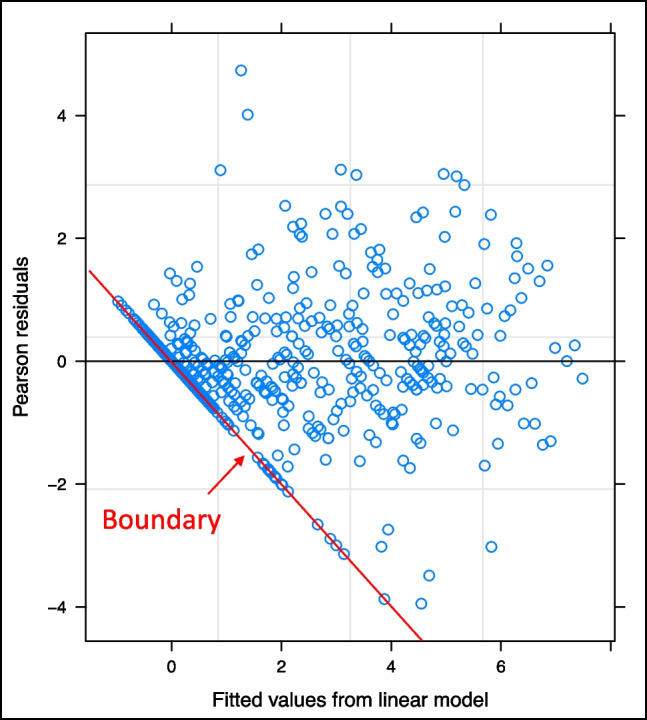
Residuals from multivariate linear model of a PRO-based neurosensory subscale score. The score demonstrates a clear boundary-value problem, demonstrating that the assumption of normality of errors inherent to *t*-tests, ANOVA, and many regression models is violated

Figure 3 shows an example of this phenomenon resulting from a multivariate regression analysis of the VHNSS. The figure shows a residual plot from a multivariate linear model. Recall that ideally the residuals would form a roughly normal distribution around the *x*-axis with no differences by fitted value. However, in this plot, the effect of the lower bound is evident in its truncation of the lower tail of the normal distribution. It is noteworthy that these boundary problems are sample size dependent. As the number of patients increases, the standard errors will shrink and the boundary will become less influential. However, in cases where the sample size cannot be increased and boundary problems are identified, analyses based on normality should be abandoned in favor of ordinal models that do not require that assumption.

#### Non-linearity

Perhaps the most obscure issue with the use of PRO measures is the fact that they do not scale linearly with respect to the latent construct they are meant to measure. This shortcoming can be illustrated with a simple clinical example. Consider a pain score on the scale from 0 to 10. There is no reason to believe that the difference in pain experienced by two patients with pain scores of zero and three is the same as the difference in pain experienced by two patients whose scores are seven and ten. In many populations, it would be common to find that the difference in pain between seven and ten would be enormous compared to the difference between zero and three. This can be seen empirically in Fig. 1, where we can see that the Rasch analysis of patients undergoing head and neck cancer therapy suggests that differences in true pain levels of those that endorse items toward the middle of the scale can be relatively small compared to the extremes. In addition, one can see that category 9 is nearly inconsequential at any pain value suggesting that the higher end of 8 and the lower end of 10 represent very similar patient experiences.

The fact that PROs do not scale linearly with the latent constructs they intend to measure has implications for how PRO measures should be analyzed [12]. The issue stems from the fact that many of the usual metrics we wish to measure in a randomized trial require that we add observations up (for example a difference in means between the treatment and control groups). By calculating the mean PRO score within a group, the analyst has made the implicit assumption that the distances above and below that mean are comparable [13, 14], i.e., if the calculated mean is 2.5 the implicit assumption is that a 1 and a 4 are equidistant from that mean value. Figure 1 illustrates that this is not always the case. Analyses that attempt to estimate a difference in means on the PRO scale are not representative of a difference in means on the latent construct scale, and often are more appropriately addressed with ordinal models that do not make assumptions about the distance between levels until it can be established that assuming linearity is justified.

This unequal spacing of PRO categories also has additional implications for combining individual items into subscale scores. Suppose we have two PRO items that are well-ordered, meaning that for every patient a higher PRO value implies a higher score on the latent construct scale. If these items are combined through addition (or averaging), there is no guarantee that the resulting subscale score will be similarly well-ordered [15].

## Conclusions

PROs are an integral part of research into the patient experience; however, their use introduces a number of complexities into the analysis and interpretations of studies that employ them. These intricacies are not currently widely addressed in studies that make use of PRO measures making it difficult to trust the results, especially when the studies fail to detect a treatment effect. Further research and education on the proper utilization of PRO measures in clinical trials is warranted.
